# LncRNA HAND2-AS1 suppressed the growth of triple negative breast cancer via reducing secretion of MSCs derived exosomal miR-106a-5p

**DOI:** 10.18632/aging.202148

**Published:** 2020-12-03

**Authors:** Li Xing, Xiaolong Tang, Kaikai Wu, Xiong Huang, Yi Yi, Jinliang Huan

**Affiliations:** 1 Department of Obstetrics and Gynecology, Shanghai Eighth People Hospital, Xuhui, Shanghai, China; 2 Department of General Surgery, Shanghai Eighth People Hospital, Xuhui, Shanghai, China

**Keywords:** triple negative breast cancer, exosome, miR-106a-5p, MSCs, HAND2-AS1

## Abstract

Background: Triple-negative breast cancer (TNBC) is a special type of breast cancer, its tumor cell metastasis rate is much higher than other types, and at the same time has a high rate of postoperative recurrence, which significantly threatens the health of women. Thus, it is urgent to explore a new treatment for TNBC.

Results: MiR-106a-5p was up-regulated in TNBC tissues and cells, and was positively correlated with the tumor grade, which indicated poor prognosis in TNBC patients. Mesenchymal stem cells (MSCs) can transport miR-106a-5p into TNBC cells via exosomes. Functional analysis showed exo-miR-106a-5p secreted by MSCs promoted tumor progression in TNBC cells. Furthermore, lncRNA HAND2-AS1 inhibited miR-106a-5p levels, and HAND2-AS1 was decreased in TNBC tissues and cells. Besides, overexpression of HAND2-AS1 reduced the secretion of exo-miR-106a-5p secretion from MSCs, thus suppressed TNBC development.

Conclusion: Our study revealed that HAND2-AS1 inhibited the growth of TNBC, which were mediated by the inhibitory effects of MSC-derived exosomal miR-106a-5p.

## INTRODUCTION

Triple negative breast cancer (TNBC) refers to breast cancer with negative expression of estrogen receptor, progesterone receptor and human epidermal growth factor receptor 2 [1]. TNBC is characterized by a poor prognosis, high recurrence and metastasis rate, and high mortality, and has become the focus of breast cancer research in recent years [2]. Because TNBC patients lack the expression of 3 receptors mentioned above, they cannot benefit from endocrine therapy and targeted therapy against human epidermal growth factor receptor 2 [3]. Thus, it is urgent to seek a new treatment for TNBC.

Exosome is a kind of cystic vesicle with a double-layer membrane structure, with a diameter of 30~100 nm. And exosomes originate from the late intracellular body of the endocytosis system [4]. It was first discovered by PAN et al. [5] in the study of extracellular cytoplasmic fusion of reticulocyte vesicles. Exosomes contain a variety of bioactive substances such as proteins, cytokines, transcription factor receptors, mRNAs, miRNAs and lncRNAs [6]. Exosomes promote intercellular communication by transporting these small molecules and become essential participants in intercellular connection under normal physiological and pathological conditions. Moreover, cells can produce different exosomes containing different genetic information under various physiological and pathological conditions [7]. Although the mechanism by which this genetic information is sorted into exosome contents is still not fully understood, it is inevitable that specific gene sequences are involved in sorting and regulating the positioning of miRNA molecules in exosomes [8]. At the same time, it can be determined that exosomes can transport these components to specific receptor cells and play particular functions, including participating in the occurrence and development of tumors [9].

Mesenchymal stem cells (MSCs) are pluripotent stem cells that can be induced to differentiate into many types of tissue cells [10]. Studies have shown that MSCs can be induced to differentiate into specific cells, such as cardiomyocytes and adipocytes. Since MSCs have the ability of immune regulation and migration [11], many studies have pointed out the role of MSCs in tumor development. MSCs used as carriers for targeted treatment of tumor cells can reach the tumor or inflammation and produce therapeutic factors to play a role in tumor suppression [12], which provides a potential new research has found that in recent years, MSCs role in regulating tumor can also be mediated by paracrine secrete outside body. It has showed that MSCs can secreted exosomes containing microRNA-144, which then inhibited the growth of tumor cells in non-small cell lung cancer [13].

MiRNA is a naturally non-coding RNA a length of about 22 nt [14]. Despite the presence of high concentrations of extracellular RNA enzymes, extracellular miRNAs have high concentrations and structural integrity in body fluids, suggesting that extracellular miRNAs may be encapsulated in a particular structure to prevent digestion by enzymes [15]. Exosomes can transport miRNAs to specific cells for specific functions, including regulating the growth of tumor cells. Studies have found that melanoma cells release small molecules including miR-214-3p and miR-199a-3p into tumor microenvironment for information communication and regulation of the development of melanoma through exosome encapsulation [16]. miR-106a-5p is a conserved miRNA involved in a variety of cancer processes [17]. And miR-106a-5p promoted ovarian cancer progression by targeting ARHGAP24, while lncRNA HOTAIRM1 sponged miR-106a-5p and inhibited ovarian cancer development [18]. However, whether miR-106a-5p can be packaged into exosomes derived from MSCs and participated in the progression of TNBC remains unclear.

Present study aimed to reveal the role of exosomal miR-106a-5p in triple TNBC process, and further investigating the underlying mechanisms.

## RESULTS

### Elevation of miR-106a-5p indicated a poor prognosis in TNBC tissues and cells

**Figure 1:**
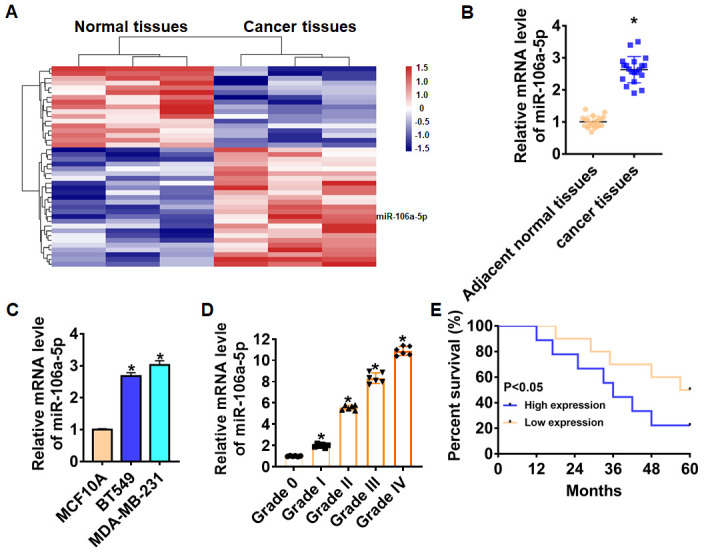
**Expression of miR-106a-5p in TNBC tissue and cells.** (**A**) MiRNAs expression profiles in normal tissues and cancer tissues of TNBC. (**B**) The expression of miR-106a-5p in clinical TNBC tissues (n = 20) and adjacent normal tissues (n = 20) determined by qRT-PCR (*p<0.05). (**C**) qRT-PCR assay analyzed the expression of miR-106a-5p in normal breast cell MCF10A and TNBC cell lines BT549 and MDA-MB-231 (*p<0.05 vs MCF10A). (**D**) The overall survival of TNBC patients with low (n = 10) or high (n = 9) expression of miR-106a-5p were assessed by Kaplan-Meier survival analysis (*p<0.05). (**E**) The expression of miR-106a-5p in TNBC tissues from patients with tumor grade 0 to grade IV (n = 6) was measured by qRT-PCR (*p<0.05 vs grade 0). The above measurement data were expressed as mean ± standard deviation. Data among multiple groups were analyzed by one-way ANOVA, followed by a Tukey post hoc test. The experiment was repeated in triplicate.

We first performed bioinformatic analysis, and the data showed the differentially expressed lncRNAs in normal and cancer tissues (Figure 1A). Then, we collected cancerous and adjacent normal tissues from 20 TNBC patients, the characteristics of patients were shown in Table 1. And qRT-PCR analysis showed that miR-106a-5p was up-regulated in cancer tissues (Figure 1B). We also found that miR-106a-5p was increased in TNBC cell lines than that in normal breast cell MCF10A (Figure 1C). According to the median level of miR-106a-5p in Figure 1A, 20 TNBC patients was divided into low (n = 10) and high expression group (n = 10). Kaplan-Meier curves indicated 5-year survival rate of TNBC patients was significantly higher in low expression patients than high expression patients (Figure 1D). Furthermore, we collected TNBC tissues from different grades (grade 0 to grade IV, n = 6) of TNBC, and found a positive correlation between miR-106a-5p level and tumor grade (Figure 1E).

**Table 1:** Clinical characteristics of TNBC patients.

**Characteristics**	**n**	**Percentage (%)**
Age		
≤50	11	55.0
> 50	9	45.0
Tumor stage		
I-II stage	5	25.0
III-IV stage	15	75.0
Lymphatic metastasis		
Positive	6	30.0
Negative	14	70.0
Tumor size		
≤5 cm	9	45.0
>5 cm	11	55.0
Distant metastasis		
M0	14	70.0
M1	6	30.0

### miR-106a-5p was packaged into exosomes and derived from MSCs

**Figure 2:**
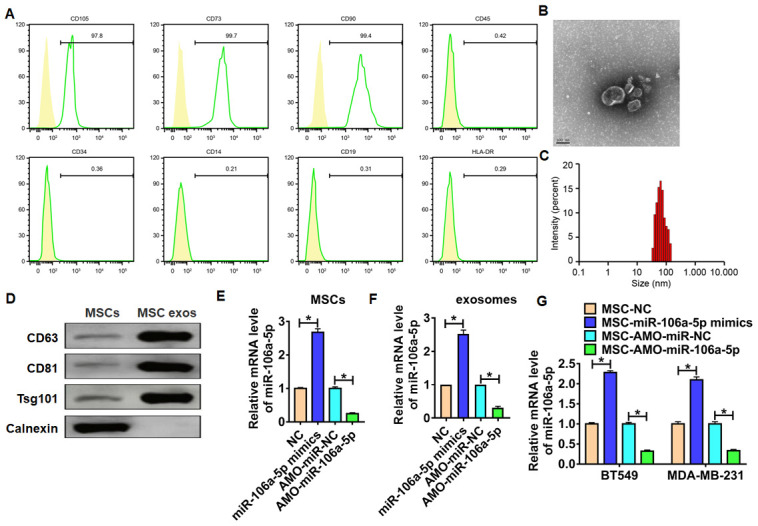
**Isolation and identification of MSCs and MSC-derived exosomes.** (**A**) Expression of BMMSC surface markers determined by flow cytometry. (**B**) TEM image for MSC-derived exosomes, scale bar = 100 nm. (**C**) Particle distribution of MSC-derived exosomes analyzed by Zetasizer Nano ZS. (**D**) Expression of exosome markers measured by western blot analysis. (**E**) miR-106a-5p expression in MSCs in response to miR-106a-5p mimic/ AMO-miR-106a-5p transfection as detected by qRT-PCR. n = 6, *p<0.05. (**F**) Exosomes in MSCs were isolated, and miR-106a-5p expression was detected using qRT-PCR. n = 6, *p<0.05. (**G**) TNBC cell lines BT549 and MDA-MB-231 cells were incubated with MSCs in response to miR-106a-5p mimic/ AMO-miR-106a-5p transfection, and miR-106a-5p expression was determined by qRT-PCR. n = 6, *p<0.05. The above measurement data were expressed as mean ± standard deviation. Data among multiple groups were analyzed by one-way ANOVA, followed by a Tukey post hoc test. The experiment was repeated in triplicate.

To identify the origin of miR-106a-5p in TNBC, we isolated MSCs from bone marrow. And flow cytometry showed that cells were positive to CD105, CD73 and CD90, but negative to CD45, CD34, CD14, CD19 and HLA-DR (Figure 2A). Next, we isolated exosomes from MSCs, and TEM showed an oval membranous vesicular disc structure (Figure 2B). Zetasizer Nano ZS showed the diameter of exosomes was about 65 nm (Figure 2C). Exosomes markers were detected by western blot (Figure 2D). To clarify the effect of MSC-derived exosomal miR-106a-5p in TNBC, we transfected miR-106a-5p mimic/AMO-miR-106a-5p MSCs. qRT-PCR showed the transfection efficiency (Figure 2E). Furthermore, exosomes were isolated from MSCs after transfection, and miR-106a-5p was also increased in isolated exosomes (Figure 2F). Then, BT549 and MDA-MB-231 cells were incubated with isolated exosomes. Interestingly, miR-106a-5p was induced in TNBC cells incubated with exosomes isolated from MSCs transfected with miR-106a-5p mimic, while miR-106a-5p was reduced in BT549 and MDA-MB-231 cells incubated with exosomes isolated from MSCs transfected with AMO-miR-106a-5p (Figure 2G). This result suggested MSCs can transport miR-106a-5p into TNBC cells via exosomes.

### Exosomal-miR-106a-5p accelerated cancer progression of TNBC cells

**Figure 3:**
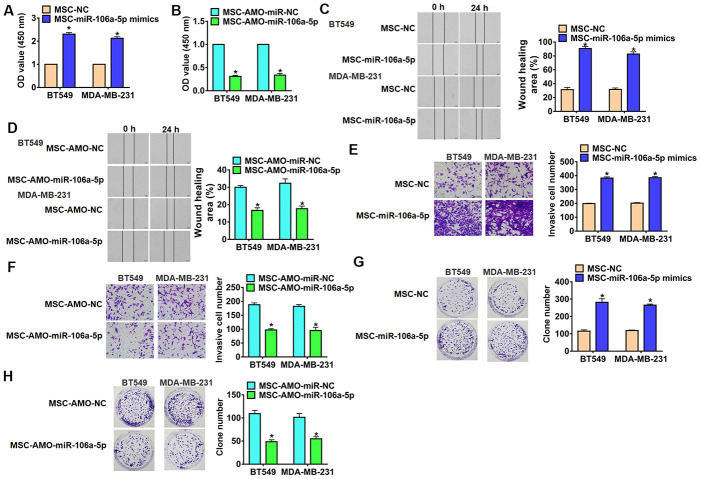
**Exo-miR-106a-5p derived from MSCs promoted migration, invasion and proliferation of TNBC cells.** BT549 and MDA-MB-231 cells were incubated with exosomes from MSCs transfected miR-106a-5p or AMO-miR-106a-5p or its NC. (**A**, **B**) MTT was used to test viability of BT549 and MDA-MB-231 cells. n = 10, *p<0.05 vs MSC-miR-NC or MSC-AMO-miR-NC. (**C**, **D**) Wound healing assay to detect migration ability. n = 4, *p<0.05 vs MSC-miR-NC or MSC-AMO-miR-NC. (**E**, **F**) Transwell assay to detect invasion ability. n = 4, *p<0.05 vs MSC-miR-NC or MSC-AMO-miR-NC. (**G**, **H**) Clone formation assay to detect proliferation ability. n = 4, *p<0.05 vs MSC-miR-NC or MSC-AMO-miR-NC. The above measurement data were expressed as mean ± standard deviation. Data among multiple groups were analyzed by one-way ANOVA, followed by a Tukey post hoc test. The experiment was repeated in triplicate.

To evaluate the role of exosomal-miR-106a-5p (exo-miR-106a-5p) in TNBC development, TNBC cells were incubated with MSCs transfected miR-106a-5p/AMO-miR-106a-5p. Functionally, we performed MTT assay to estimate cell viability. It showed that exo-miR-106a-5p increased cell viability (Figure 3A), while AMO-miR-106a-5p decreased cell viability (Figure 3B). Furthermore, wound healing assay suggested that exo-miR-106a-5p from MSCs promoted cell migration in TNBC cells (Figure 3C), exo-AMO-miR-106a-5p showed an opposite effect (Figure 3D). Transwell assay showed that exo-miR-106a-5p from MSCs induced cell invasion in TNBC cells (Figure 3E), but exo-AMO-miR-106a-5p inhibited invasive ability (Figure 3F). In addition, exo-AMO-miR-106a-5p promoted proliferation of TNBC cells (Figure 3G), while silencing miR-106a-5p inhibited proliferative ability (Figure 3H). Together, exo-miR-106a-5p secreted by MSCs promoted tumor progression in TNBC cells.

### LncRNA HAND2-AS1 inhibited miR-106a-5p through ceRNA

**Figure 4:**
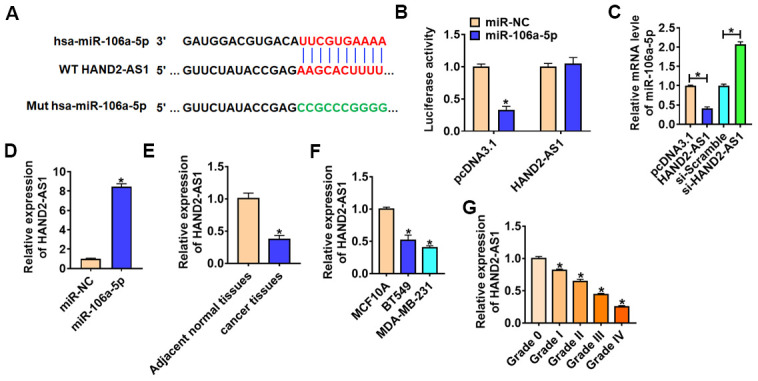
**HAND2-AS1 inhibited miR-106a-5p expression.** (**A**) miRanda database predicted data between HAND2-AS1 and miR-106a-5p. (**B**) Luciferase assay for WT and mutant HAND2-AS1 activity in HEK293 cells transfected with miR-NC or miR-106a-5p. n = 6, *p<0.05. (**C**) qRT-PCR analyzed the expression of miR-106a-5p in BT549 cells transfected with HAND2-AS1 or si-HAND2-AS1. n = 6, *p<0.05. (**D**) RNA-immunoprecipitation experiments were performed using miR-NC or miR-106a-5p to immunoprecipitate HAND2-AS1 in BT549 cells. (**E, F**) qRT-PCR analyzed HAND2-AS1 expression in TNBC tissues and cells. n = 6, *p<0.05. (**G**) The expression of HAND2-AS1 in TNBC tissues from patients with tumor grade 0 to grade IV (n = 6) was measured by qRT-PCR (*p<0.05 vs grade 0). The above measurement data were expressed as mean ± standard deviation. Data among multiple groups were analyzed by one-way ANOVA, followed by a Tukey post hoc test. The experiment was repeated in triplicate.

Accumulating evidence showed lncRNAs sponged miRNAs and inhibited the expression and activity of miRNAs in many diseases, especially cancers. MiRanda database showed there were paired bases between HAND2-AS1 and miR-106a-5p (Figure 4A). Then, we performed dual-luciferase reporter assay in HEK293 cell line, and found that the luciferase activity of WT HAND2-AS1, but not mutant HAND2-AS1, was significantly repressed in the miR-106a-5p group (Figure 4B). Furthermore, qRT-PCR analysis showed overexpression of HAND2-AS1 significantly inhibited miR-106a-5p level, while si-HAND2-AS1 promoted miR-106a-5p expression (Figure 4C). And there was a significant enrichment of HAND2-AS1 bound to miR-NC comparing with the miR-106a-6p in BT549 cells (Figure 4D). In addition, HAND2-AS1 was significantly decreased in TNBC tissues and cells (Figure 4E and 4F). And HAND2-AS1 expression was negatively correlated with the tumor grade (Figure 4G).

### Overexpression of HAND2-AS1 suppressed TNBC development by inhibiting exo-miR-106a-5p secretion from MSCs

**Figure 5:**
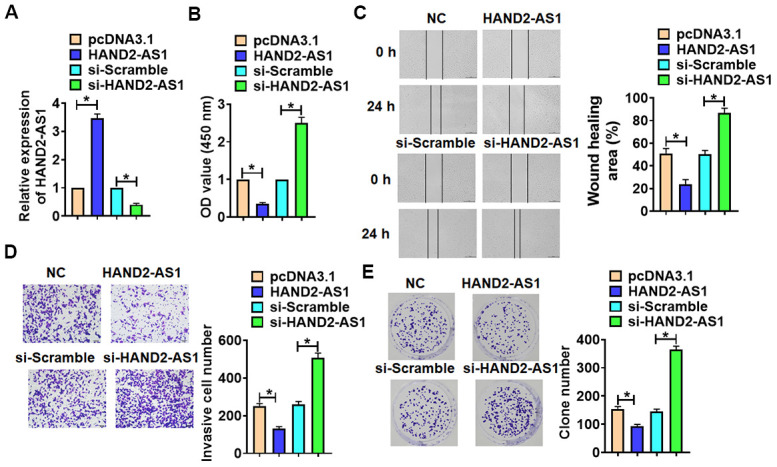
**The effects of HAND2-AS1 on the proliferation, migration and invasion of TNBC cells.** (**A**) HAND2-AS1 or si-HAND2-AS1 was transfected into BT549 cells, and transfection efficiency of HAND2-AS1 or si-HAND2-AS1 was detected using qRT-PCR. n = 6, *p<0.05. (**B**) MTT assay for BT549 cells. n = 10, *p<0.05. (**C**) would healing assay was to detected migration of BT549 cells. n = 4, *p<0.05. (**D**) Transwell assay was to determine invasion of BT549 cells. n = 4, *p<0.05. (**E**) Clone formation assay was to evaluate proliferation of BT549 cells. n = 4, *p<0.05. The above measurement data were expressed as mean ± standard deviation. Data among multiple groups were analyzed by one-way ANOVA, followed by a Tukey post hoc test. The experiment was repeated in triplicate.

To confirm the effects of HAND2-AS1 on TNBC progression, we forced or silenced HAND2-AS1 expression in BT549 cells (Figure 5A). Then, we performed functional analysis and found that overexpression of HAND2-AS1 suppressed cell viability, migration, invasion and proliferation, while deletion of HAND2-AS1 showed a opposite effects (Figure 5B, 5E).

**Figure 6:**
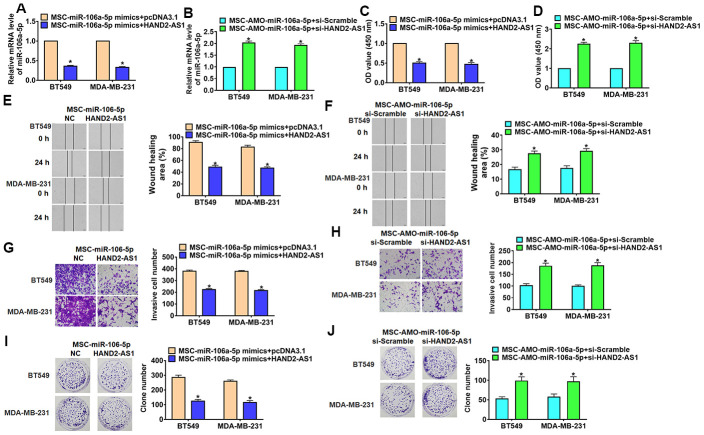
**Overexpression lncRNA HAND2-AS1 inhibited progression of TNBC cells by regulating exo-miR-106-5p.** BT549 and MDA-MB-231 cells were transfected with HAND2-AS1 or si-HAND2-AS1 and incubated with exosomes from MSCs transfected miR-106a-5p or AMO-miR-106a-5p. (**A**, **B**) qRT-PCR analyzed miR-106a-5p expression in BT549 and MDA-MB-231 cells. n = 6, *p<0.05. (**C**, **D**) MTT for BT549 and MDA-MB-231 cells. n = 10, *p<0.05. (**E**, **F**) Migrative ability was detected by wound healing assay. n = 4, *p<0.05 vs MSC-miR-NC or MSC-AMO-miR-NC. (**G, H**) Invasive ability was detected by Transwell assay. n = 4, *p<0.05. (**I**, **J**) Proliferative ability was detected by clone formation assay. n = 4, *p<0.05. The above measurement data were expressed as mean ± standard deviation. Data among multiple groups were analyzed by one-way ANOVA, followed by a Tukey post hoc test. The experiment was repeated in triplicate.

In order to verify whether miR-106a-5p is the downstream molecule of HAND2-AS1 in TNBC process, TNBC cells were transfected with HAND2-AS1 or si-HAND2-AS1 and incubated with MSCs transfected with miR-106a-5p/ AMO-miR-106a-5p, respectively. qRT-PCR assay showed the transfection efficiency of HAND2-AS1 or si-HAND2-AS1 in TNBC cells (Figure 6A). As well, overexpression of HAND2-AS1 inhibited miR-106a-5p expression, while si-HAND2-AS1 promoted miR-106a-5p expression in TNBC cells (Figure 6B). Followed experiments indicated that HAND2-AS1 reduced cell viability, migration, invasion and proliferation in TNBC cells (Figure 6C, 6E, 6G, 6I), while silencing of HAND2-AS1 showed the opposite function (Figure 6D, 6F, 6H, 6J). Thus, HAND2-AS1 inhibited secretion of exo-miR-106a-5p from MSCs and then inhibited TNBC development.

### HAND2-AS1 inhibited in vivo tumor growth in the nude mice by downregulating miR-106a-5p

**Figure 7:**
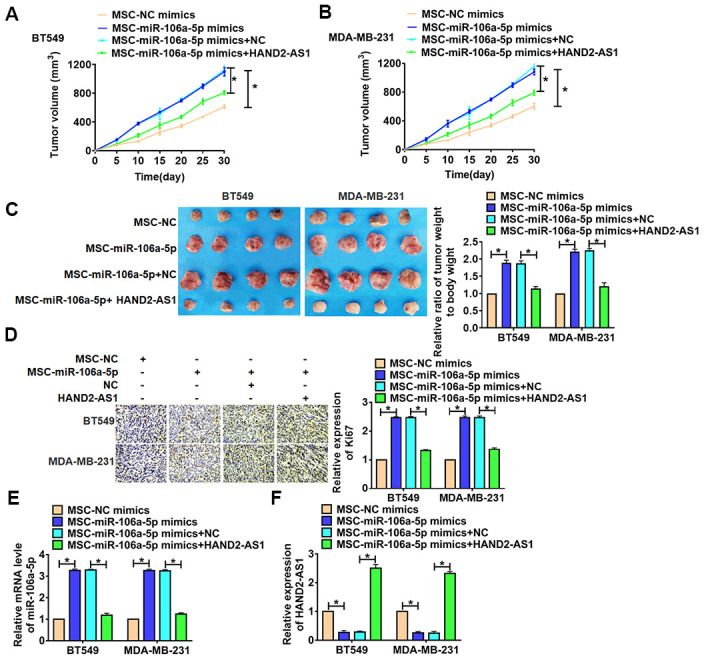
**HAND2-AS1 inhibited TNBC tumorigenesis in vivo. BT549 and MDA-MB-231 cells were incubated with exosomes from MSCs transfected with miR-106a-5p, then were injected into nude mice.** 1 week later, lentivirus packaging HAND2-AS1 was injected into tumors. (**A**,.**B**) Growth of tumor xenografts in nude mice. n = 6, *p<0.05. (**C**) Representative tumors excised from xenografts in nude mice and tumor weight, and the ratio of tumor weight to body weight was calculated. n = 6, *p<0.05. (**D**) Expression of Ki67 by immunohistochemical staining in tumors. n = 6, *p<0.05. (**E**, **F**) The expression of miR-106a-5p and HAND2-AS1 in tumors were detected by qRT-PCR. n = 6, *p<0.05. The above measurement data were expressed as mean ± standard deviation. Data among multiple groups were analyzed by one-way ANOVA, followed by a Tukey post hoc test. The experiment was repeated in triplicate.

For further explore the function of HAND2-AS1 in TNBC, we set up xenograft nude mice model. TNBC cells were incubated with exosomes from MSCs transfected with miR-106a-5p, then were injected into nude mice. 1 week later, lentivirus packaging HAND2-AS1 was injected into tumors. Tumors grew faster and bigger in the mice with MSCs-miR-106a-5p, while injection of HAND2-AS1 inhibited the growth rate and volume of tumors (Figure 7A, 7B). The tumors were isolated at 30 days after injection, MSCs-miR-106a-5p significantly increased tumors weight, and HAND2-AS1 removed the promoting role of MSCs-miR-106a-5p (Figure 7C, 7D). In addition, immunohistochemical assay showed MSCs-miR-106a-5p induced the expression of Ki67, while HAND2-AS1 reduced Ki67 level (Figure 7E, 7F). Then, qRT-PCR showed injection of HAND2-AS1 increased HAND2-AS1 level in tumors. And MSCs-miR-106a-5p increased miR-106a-5p expression in tumors, while overexpression of HAND2-AS1 inhibited miR-106a-5p expression. Thus, HAND2-AS1 inhibited TNBC growth by downregulating miR-106a-5p.

## DISCUSSION

TNBC is a special type of breast cancer, its tumor cell metastasis rate is much higher than other types, and at the same time has a high rate of postoperative recurrence, which greatly threatens the health of women [19]. Although researchers continue to explore the treatment of TNBC and related drugs, so far there is no effective targeted therapy [20]. Thus, many researchers began to actively seek therapeutic targets at the molecular level.

At present, the extracellular miRNAs have been detected in various physiological and pathological conditions. A large number of miRNAs can be released simultaneously and regulate multiple targets, thereby activating a complex network of transduction [21]. These miRNAs are secreted from exosomes into the microenvironment, and after binding with the corresponding receptor cells, they act on the target mRNA to change the protein expression and perform biological functions [22]. Meanwhile, certain expression patterns have been found in a variety of tumor patients, making them a new class of tumor biomarkers and therapeutic targets in recent years. Rabinowits et al. [23] used exosomes as genetic material for the first time, and analyzed the differences between exosomal miRNAs in normal human serum and miRNAs in NSCLC patients' serum. As well, there are many specific miRNAs in exosomes of patients with triple negative and Her2 positive breast cancer [24]. In present study, we found that miR-106a-5p was increased in TNBC tissues and cells, and the expression of miR-106a-5p was positively correlated with the tumor grade, which indicated a poor prognosis in TNBC patients. This finding was similar with previous researches targeting on miR-106a-5p and tumor, which also showed an elevation of miR-106a-5p in gastric cancer [25] and hepatocellular carcinoma [26].

When a tumor occurs in the body, MSCs can immediately sense the call of abnormal signals and gather in the tumor microenvironment [27]. Exosomes secreted by MSCs can carry a large amount of proteins and genetic information, and regulate the growth of tumor cells by transferring the therapeutic genes to the target cells [28]. Under the comprehensive influence of multiple factors, the regulation effect of MSC-derived exosomes on tumor is different, mainly presenting as promoting or inhibiting effect [29]. And in present study, we speculated that miR-106a-5p may be packaged into exosomes and secreted by MSCs. Thus, we isolated MSCs from bone marrow of TNBC patients, and further isolated exosomes from MSCs. Then TNBC cells were co-cultured with exosomes from MSCs transfected with miR-106a-5p or AMO-miR-106a-5p. Interestingly, MSCs transfected with miR-106a-5p promoted miR-106a-5p expression in TNBC cells, while MSCs transfected with AMO-miR-106a-5 inhibited miR-106a-5p expression. These data indicated miR-106a-5p can be packaged into exosomes and secreted into TNBC cells by MSCs. followed functional experiment showed exo-miR-106a-5p promoted TNBC development. However, our present data only detected MSCs, exo-miR-106a-5p maybe secreted by cancer stem cells, which will be determined in our further study.

At present, numerous studies have shown that lncRNA participates in a variety of physiological and pathological processes through the ceRNA mechanism regulating miRNAs [30]. Herein, we predicted that a binding between miR-106a-5p and HAND2-AS1. And it has been reported that HAND2-AS1 was involved in the progression of breast cancer [31, 32]. And our results showed HAND2-AS1 acted as a sponge of miR-106a-5p, and HAND2-AS1 was decreased in TNBC tissues and cells. In addition, overexpression of HAND2-AS1 inhibited the secretion of exo-miR-106a-5p secretion from MSCs, thus suppressed TNBC development both in vitro and in vivo. However, present study only focused HAND2-AS1 and exo-miR-106a-5p function in TNBC development, the underlying mechanism will be further explored in our future study. In addition, we will try to make a specific nanoparticle of HAND2-AS1 and to cue TNBC.

Exosomes, as transport carriers of small molecules in vivo, have their unique advantages over traditional transport methods. Firstly, exosomes are derived from endogenous cells and are a natural carrier with good immunogenicity. Exosomes rarely cause toxicity and immune responses when they are used in organism. Secondly, exosomes with a diameter of only 30 ~ 100 nm are an ideal way to transport miRNA without being phagocytized by macrophages. And exosomes can specifically transport anti-tumor or pro-tumor miRNA antisense sequences to the target sites so as to regulate protein expression. Therefore, as the transport carrier of miRNA, exosomes have a certain practical application prospect, which is conducive to the transformation of basic research of miRNA into clinical application.

## CONCLUSIONS

In conclusion, our study revealed exo-miR-106a-5p secreted by MSCs promoted TNBC progression, which can be inhibited by lncRNA HAND2-AS1. And our study provided an attractive target in TNBC clinical treatment.

## MATERIALS AND METHODS

### Clinical samples

Cancerous and normal tissues were taken from 20 TNBC patients January 2018 to January 2019 (Table 1). And TNBC tissues were collected from different grades: grade 0 to grade IV (n = 6)). All of the patients or their guardians provided written consent, and the Ethics Committee of Shanghai Eighth People Hospital approved all aspects of this study.

### MSCs and exosome isolation and identification

MSCs were isolated from bone marrow of TNBC patients as previous description [13], which identified with flow cytometry. Several centrifugations were performed to purify exosomes in culture medium. Transmission electron microscopy (TEM) was used to identify exosomes structures. MSCs-derived exosomes were analyzed using exosome marker protein CD63, CD81, Tsg101 and Calnexin via Western blot. Exosomes size was calculated using Zetasizer Nano ZS.

### Cell culture and treatment

The BT549 and MDA-MB-231 (Science Cell Laboratory) were cultured in RPMI 1640 supplemented with 10 % fetal bovine serum and 100 μL/mL penicillin and streptomycin. 500 nM miR-106a-5p mimics or 500 nM AMO- miR-106a-5p or 2 μg HAND2-AS1 plasmid or its NC was transfected into cells with Lipo 2000, respectively. 5ug/ml exosomes were added into the medium of BT549 and MDA-MB-231 cells every 24 h. Plasmid of HAND2-AS1 or small interfering RNA (si-RNA) of HAND2-AS1 or miR-106a-5p mimics or AMO- miR-106a-5p were constructed by Genechem (Shanghai, China).

### qRT-PCR

Total RNA was isolated from serum and culture medium according to a standard protocol. And then, the purity and concentration of RNA was detected and all the samples were converted into cDNA using reverse transcription kit. We used SYBR Green (Thermo Fisher Scientific, USA) system to perform the qRT-PCR. Data was analyzed by GraphPad 7.0 Primer lists: HAND2-AS1 (F: 5′-GGGTGTTTACGTAGACCAGAACC-3′, R: 5′-CTTCCAAAAGCCTTCTGCCTTAG-3′), β-actin (F: 5′-TCACCCACACTGTGCCCATCTACGA-3′, R: 5′-CAGCGGAACCGCTCATTGCCAATGG-3′), miR-106a-5p (F: 5'-GATGCTCAAAAAGTGCTTA CAGTGCA-3', R: 5'-TATGGTTGTTCTGCTCTCTGTCTC-3'), U6 (F: 5′-CGGAATTCCCCCAGTGGAAAGACGCG CAG-3′, R: 5′-CGGTGTTTCGTCCTTTCCACAAG-3′).

### Western blot

Protein samples were blotted depended on standard protocol. And we used Odyssey Infrared Scanning System (Gene Co. Ltd., Hongkong, China) to scan the membranes. At last, we used Image J software to analyze the western bolt results. The primary antibodies are as list: CD63 (CBL553), CD81 (SAB3500454), Tsg101 (SAB2109010) and Calnexin (SAB4503258) were purchased from Sigma-Aldrich. The secondary antibodies IRDye700/800 Mouse or Rabbit were produced by LICOR (Lincoln, NE, USA).

### MTT assay

Cells were plated in 96-well plates and we used MTT assay to detect the cell viability. MTT (0.5 mg/mL; Beyotime Biotechnology, China) was added into cells and incubated for 4 h in incubator. We measured the absorbance of cell in 150 μL DMSO by Spectrophotometer (Tecan, Austria) at 450 nm.

### Immunohistochemistry assay

Frozen sections of tumors were fixated in 4% paraformaldehyde and washed using PBS. We penetrated sections using 0.5% Triton X-100. After 3 times wash, we blocked sections with 50% goat serum. Then, sections were incubated with Ki67 antibody overnight. Then, we incubated the sections using secondary antibody. Immunofluorescence was analyzed under an IX73 fluorescence microscope (Olympus, Valley, PA).

### Wound healing assay

The cells were spread in a 6-well plate, and when they grew to 80%, horizontal lines were drawn in the cells with a ruler at an interval of 0.5cm, with 4 lines drawn in each well. The cells were washed with PBS for 3 times. Serum free culture medium was added and photographed at 0 hours. The cells were continuously cultured for 24 hours before the photo was taken.

### Transwell assay

First, the Matrigel was spread over the Transwell, and the starved cells for 12 hours were inoculated into the upper chamber. The culture medium with serum was added to the lower chamber and cultured for 12 hours. The Transwell was taken out, the culture medium was discarded. And Transwell was washed using PBS, and fixed with methanol for 30 minutes. After the chamber was dried, the cells were stained with crystal violet for 20min, and the upper cells were removed and washed with PBS for 3 times. The cells were photographed and counted under the microscope.

### Clone formation assay

Cells were seeded into 6 well plates at a density of 100 cells/well and cultured for 14 days. Cells were fixed with 4% and stained with 0.1% Crystal Violet (Sigma-Aldrich, USA) at RT for 15min. Then cells were rinsed with distilled water, and the colonies were visualized by inverted microscope.

### RNA-Binding Protein Immunoprecipitation (RIP)

We performed a RIP assay to determine the binding between HAND2-AS1 and miR-106a-5p using Magna RIP™ RNA-Binding Protein Immunoprecipitation Kit (Millipore) as previous study. Briefly, BT549 cells were transfected with biotinylated miR-106a-5p, and the expression of HAND2-AS1 was detected using qRT-PCR.

### Animal experiment

BT549 and MDA-MB-231 cells were injected into nude mice (Guangdong provincial experimental animal center). And exosomes were isolated from MSCs transfected with miR-106a-5p or NC, then a dosage of 5 mg exosomes was administered into mice via tail vein injection once every 3 days for 2 weeks. 1 week later, lentivirus packaging HAND2-AS1 was injected into tumors. And tumor size was measured every 5 days. After 30 days of injection, mice were intraperitoneally injected with 3% pentobarbital sodium and were killed by excessive intraperitoneally anesthesia with a dose of 90 mL/kg, and the tumors were removed for follow-up study.

### Statistical analysis

All data is presented as a mean ± S.E.M. Statistical analysis was performed using Student's t-test or Wilcoxon test or a one-way ANOVA through Graphpad Prism 7.0.
